# Hydrogen sulfide increases intracellular oxygen and inhibits the HIF response

**DOI:** 10.1016/j.jbc.2026.111151

**Published:** 2026-01-12

**Authors:** Joseph Brake, David A. Hanna, Roshan Kumar, Qianni Peng, Aaron P. Landry, Rashi Singhal, Eranthie Weerapana, Yatrik M. Shah, Ruma Banerjee

**Affiliations:** 1 Department of Biological Chemistry, University of Michigan, Ann Arbor, Michigan, USA; 2 Chemistry Department, Boston College, Chestnut Hill, Massachusetts, USA; 3 Department of Molecular and Integrative Physiology, Ann Arbor, Michigan, USA; 4 Department of Internal Medicine, Ann Arbor, Michigan, USA; 5 Rogel Cancer Center, University of Michigan, Ann Arbor, Michigan, USA

**Keywords:** hypoxia, hydrogen sulfide, oxygen metabolism, sulfide quinone oxidoreductase, hypoxia-inducible factor, hypoxia sensing

## Abstract

Oxygen (O_2_) sensing by hypoxia-inducible factor (HIF) is a principal mechanism by which aerobic organisms adjust cellular energy metabolism and adapt to O_2_ limitation. In this study, we show that hydrogen sulfide (H_2_S), a product of host and microbial metabolism, profoundly influences the threshold for HIF-dependent hypoxia sensing by increasing intracellular O_2_. The dose-dependent destabilization of HIF by H_2_S is inversely correlated with sulfide quinone oxidoreductase, which oxidizes sulfide in the mitochondrion. Hypoxia sensors provide a semiquantitative estimate of the magnitude of H_2_S-induced perturbation. Thus, the O_2_ concentration in cells grown in a 2% O_2_ atmosphere is sensed as 5% or 15% O_2_ in the presence of 25 or 100 ppm H_2_S, respectively. Sustained exposure to H_2_S elicits the hallmarks of hyperoxia-associated cytotoxicity, including loss of iron–sulfur proteins in cellular and murine models. H_2_S thus emerges as a powerful regulator of O_2_ sensing and signaling with possible implications for dysregulation in O_2_ toxicity diseases.

Biospheric oxygenation, which occurred ∼2.2 billion years ago, introduced a high-potential electron acceptor and substrate, fueling the evolution of new biocatalytic reactions and pathways. Today, oxygen (O_2_) is estimated to be the most used substrate across metabolomes, surpassing even ATP and NADH (1). Compared with 21% O_2_ (160 mm Hg) in the atmosphere, O_2_ levels in human tissue vary from ∼14% O_2_ (110 mm Hg) in lung alveoli to <0.1% O_2_ (<1 mm Hg) in the intestinal lumen (2, 3, 4, 5). Hypoxia-inducible factor (HIF) is a principal O_2_ sensor that activates an adaptive transcriptional program for survival in low O_2_ (6, 7, 8). Under normoxic conditions, O_2_-dependent hydroxylation of the α-subunit of HIF by prolyl hydroxylase (PHD) tags it for ubiquitination by the von Hippel–Lindau tumor-suppressor protein, a component of an E3 ligase complex, for subsequent proteasomal degradation (9, 10). An estimated 90% of whole body O_2_ consumption occurs at the electron transport chain (ETC) (11). Flux modulation and maintenance of tissue O_2_ gradients by endogenous ETC regulators are poorly understood. A mouse model for Leigh syndrome deficient in the complex I subunit Ndufs4 shows evidence of brain hyperoxia that can be reversed by hypoxia (12), highlighting the importance of ETC activity in O_2_ homeostasis.

The motivation of the current study was to investigate how H_2_S, which is a product of our own metabolism (13) and is also produced in copious quantities by gut microbes (14, 15), impacts O_2_ metabolism. In addition to being a respiratory poison that targets cytochrome *c* oxidase or complex IV (16), H_2_S is also an ETC substrate (17). In the colon, the O_2_ gradient is shaped by the complex interaction between host and microbial metabolism, and colonocytes occupy the liminal zone between a microbiota-dense and virtually anoxic lumen and a highly vascular lamina propria (18). The microbiome responds to luminal O_2_ levels, and the O_2_ gradient, in turn, affects host–microbiome interactions (2, 19). Chronic pathological hypoxia is a signature of active inflammatory bowel disease and, if unchecked, contributes to disease progression (20). H_2_S is reported to both attenuate and augment HIF stability (21, 22, 23, 24). Like nitric oxide, H_2_S reportedly induces metabolic hypoxia and redistributes O_2_ usage (21, 25).

In this study, we demonstrate that H_2_S elicits a dose-dependent destabilization of HIF under hypoxia (2% O_2_) by increasing intracellular O_2_. The magnitude of HIF destabilization is inversely correlated with expression of the H_2_S-oxidizing enzyme, sulfide quinone oxidoreductase (SQOR), and exacerbated by its knockdown. By calibrating intracellular O_2_ exposure in cells grown under ambient hypoxia and varying levels of H_2_S, we provide semiquantitative estimates of the magnitude by which H_2_S modulates the threshold for HIF-dependent hypoxia sensing. Further, while sustained H_2_S-dependent complex IV inhibition induces a reductive shift in redox cofactor pools (26), we find a concomitant oxidative shift in lipids as well as in the mitochondrial cysteine proteome, accompanied by decreased levels of iron–sulfur (Fe–S) cluster proteins, a fingerprint of hyperoxia. Our study reveals that H_2_S is a powerful regulator of intracellular O_2_ availability and HIF signaling.

## Results

### Hypoxia restores Fe–S cluster proteins destabilized by H_2_S

**Figure 1:**
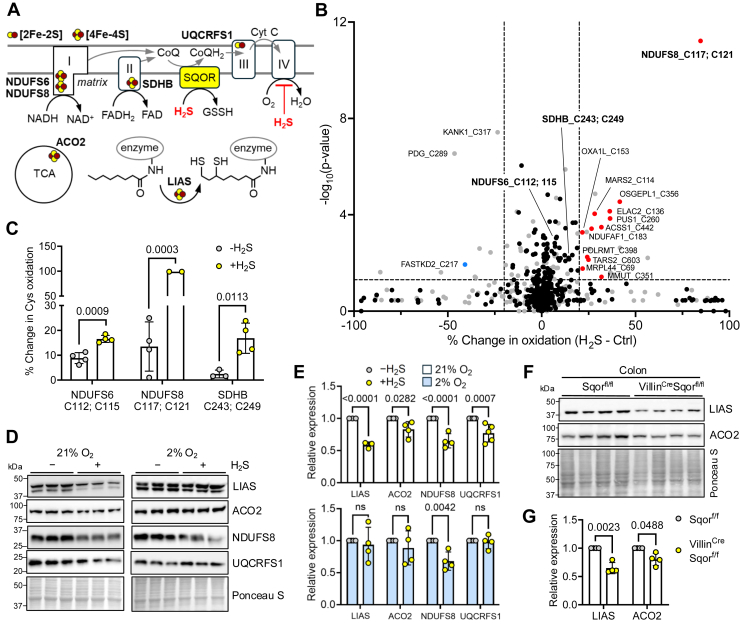
**Hypoxia restores Fe–S proteins destabilized by H_2_S.***A*, model showing metabolic changes because of complex IV inhibition by H_2_S-mediated increase in O_2_, which can be mitigated by SQOR activity. Six Fe–S proteins that were found to be destabilized in this study by H_2_S are noted. *B*, volcano plot of PL-OxICAT data displaying the percent change in oxidation for cysteines in HEK293 cells grown in the absence or presence of H_2_S (100 ppm, 24 h). The *black* and *gray dots* indicate mitochondrial and nonmitochondrial cysteines, and the *red* and *blue dots* indicate oxidized and reduced mitochondrial cysteines, respectively. *Horizontal and vertical dashed lines* indicate *p* < 0.05 and a change in oxidation of 20%, respectively. *C*, quantitation of select Fe–S cluster–coordinating cysteines from the data in *B*. *D* and *E*, representative Western blots (*D*) and quantitation (*E*) of select Fe–S proteins in HT-29 cells cultured at 21 or 2% O_2_ ± 100 ppm H_2_S (n = 3 or 4). The reference denotes protein levels in the absence of H_2_S, arbitrarily set at 1. *F* and *G*, representative Western blots (*F*) and quantitation (*G*) of colon Fe–S proteins from Sqor^fl/fl^ (n = 4) and *Villin*^Cre^ Sqor^fl/fl^ mice (n = 4). A two-way ANOVA followed by Šídák post hoc test was performed for statistical analysis; the data in *C*, *E*, and *G* represent the mean ± SD. Ponceau staining for equal loading is shown below the respective Western blots. Fe–S, iron–sulfur; HEK293, human embryonic kidney 293 cell line; H_2_S, hydrogen sulfide; PL-OxICAT, proximity labeling oxidative isotope–coded affinity tag; SQOR, sulfide quinone oxidoreductase.

Decreased forward electron flow because of complex IV inhibition has pleiotropic effects, including a reductive shift in redox cofactor pools (26, 27) and potentially, enhanced reactive oxygen species (ROS) and reactive sulfur species generation because of increased electron leakage (Fig. 1*A*). We observed increased lipid peroxidation in colon adenocarcinoma HT-29 cells exposed to H_2_S as reported by the BODIPY dye (Fig. S1, *A* and *B*). The magnitude of lipid oxidation elicited by H_2_S (100 ppm, 24 h) was comparable to that induced by cumene hydroperoxide (100 μM), employed as a positive control (Fig. S1, *C* and *D*). Next, we used the proximity labeling oxidative isotope–coded affinity tag platform (28) to evaluate how H_2_S affects the redox status of cysteine thiols in the mitochondrial matrix proteome, which we targeted because of its proximity to electron leakage from the ETC. Relative to cells grown in the absence of H_2_S, a broad oxidative shift was observed in the presence of H_2_S (100 ppm for 24 h, corresponding to ∼20 μM dissolved sulfide in the culture medium) (Fig. 1*B*, Table S1). Notably, some of the oxidatively shifted targets were Fe–S proteins and included cluster-coordinating cysteine residues in NDUFS8 and SDHB, which are subunits of complex I and II, respectively (Fig. 1*C*).

Next, we assessed the stability of mitochondrial Fe–S proteins with roles in lipid metabolism, the ETC, and the tricarboxylic acid cycle (Fig. 1*A*). Statistically significant decreases were seen in the expression of LIAS, ACO2, NDUFS8, and UQCRFS1 in HT-29 cells grown in an atmosphere of 21% O_2_ and 100 ppm H_2_S (Fig. 1, *D* and *E*). Destabilization of Fe–S proteins is a signature of hyperoxia and is rescued by hypoxia (29). We hypothesized that H_2_S-mediated inhibition of cellular respiration, leading to intracellular O_2_ accumulation, was responsible for the broad destabilization of Fe–S proteins. Remarkably, with the exception of NDUFS8, hypoxia (2% O_2_) protected against H_2_S-triggered depletion of Fe–S proteins (Fig. 1, *D* and *E*), suggesting increased synthesis and/or stabilization of metal clusters under these conditions. The physiological relevance of Fe–S protein destabilization by elevated H_2_S exposure was assessed in *Villin*^Cre^ Sqor^fl/fl^ mice that lack the capacity for SQOR-dependent sulfide oxidation in intestinal epithelial cells, which is expected to result in sustained exposure to higher levels of H_2_S (30) (Fig. 1*A*). Statistically lower LIAS and ACO2 levels were seen in the colon of *Villin*^Cre^ Sqor^fl/fl^ mice (Fig. 1, *F* and *G*) as seen in HT-29 cells, although UQCRFS1 was unchanged (Fig. S2, *A* and *B*).

### H_2_S destabilizes HIF-1α under hypoxia

**Figure 2:**
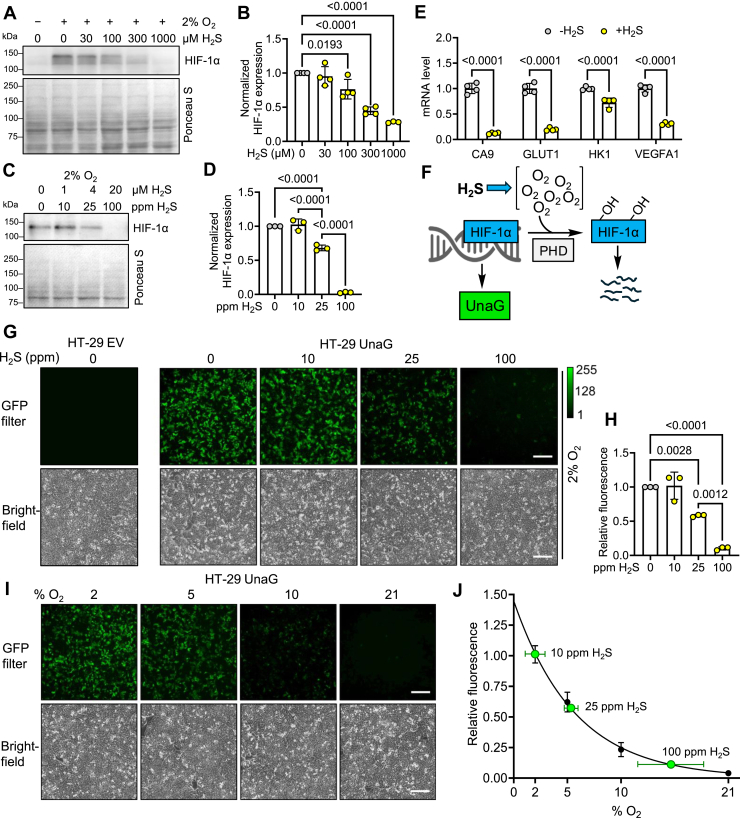
**H_2_S shifts the cellular threshold for hypoxia sensing.***A*, representative Western blot of HIF-1α in HT-29 cells grown under hypoxia (2% O_2_) with acute exposure to the indicated H_2_S concentration for 1 h (n = 3 or 4). *B*, quantitation of data in *A* normalized to Ponceau S staining for total protein per lane. *C*, representative Western blot of HIF-1α in HT-29 cells grown under hypoxia (2% O_2_) with chronic exposure to the indicated H_2_S concentration for 24 h. *D*, quantitation of data in *C* normalized to Ponceau S staining for total protein per lane (n = 3). *E*, expression of HIF target genes in HT-29 cells grown in 2% O_2_ ± 100 ppm H_2_S for 24 h normalized to the mean of three reference genes (n = 4). *F*, scheme showing activation of green fluorescence by the hypoxia sensor UnaG. *G*, effect of H_2_S (0–100 ppm) on hypoxia (2% O_2_)-induced fluorescence in UnaG-expressing HT-29 cells. *H*, quantitation of data in *G* relative to the minus H_2_S condition (n = 3). *I*, fluorescence in UnaG-expressing HT-29 cells grown in the presence of 2% to 21% O_2_. *J*, dependence of UnaG fluorescence on O_2_ concentration (normalized to 2% O_2_ = 1) and fit to an exponential decay curve (n = 3). The *green dots* correspond to fluorescence observed at the indicated concentration of H_2_S (data from *G*). The scale bar represents 200 μm in all images. A two-way ANOVA followed by Šídák post hoc test was performed for statistical analysis; the data represent the mean ± SD. HIF, hypoxia-inducible factor; H_2_S, hydrogen sulfide.

Restoration of Fe–S protein levels by shifting to a hypoxic atmosphere is consistent with our model that cluster damage results from sulfide-induced O_2_ accumulation (Fig. 1*A*). We therefore characterized the effect of H_2_S on hypoxia sensing by HIF. Acute exposure to Na_2_S (0.03–1 mM, 1 h) induced a dose-dependent decrease in HIF-1α stabilization in cells grown at 2% O_2_ (Fig. 2, *A* and *B*). Since H_2_S disappears with a *t*_1/2_ of 3 to 4 min at 37 °C (31), the difference in HIF-1α between with and without H_2_S samples was not seen beyond 1 h, which revealed reversibility of the H_2_S effect (Fig. S2, *C* and *D*). We note that although HIF-1α has a molecular mass of 92.6 kDa, two major bands with estimated masses of 120 and 130 kDa were often, but not always, detected by Western blot analysis. Under hypoxic conditions, the less intense lower band was stabilized earlier, and the difference was clearly seen in the high-exposure display (Fig. S2*C*). H_2_S also destabilized HIF-2α under hypoxia, although the magnitude of the effect was smaller relative to HIF-1α because of high HIF-2α expression under normoxia (Fig. S2, *E* and *F*). We therefore focused on HIF-1α as a marker to characterize changes in O_2_ levels by H_2_S. H_2_S destabilized HIF-1α in EA.hy296, HCT116, and human embryonic kidney 293 (HEK293) cells (Fig. S2, *G*–*L*).

A limitation of bolus Na_2_S treatment is that the concentration of H_2_S is unstable over the course of the experiment because of its volatilization and metabolism. To circumvent this drawback, cells were cultured in a custom growth chamber with an H_2_S atmosphere to mimic chronic exposure experienced by colonocytes (32). The H_2_S concentration was varied between 0 and 100 ppm for 24 h, which corresponds to a dissolved sulfide concentration in the culture medium of 0 to 20 μM and was shown not to affect cell viability (32). A statistically significant decrease in HIF-1α was observed at 25 ppm H_2_S (4 μM dissolved sulfide), and HIF-1α stabilization was completely abolished at 100 ppm H_2_S (20 μM dissolved sulfide) (Fig. 2, *C* and *D*). RT–quantitative PCR (qPCR) analysis revealed lower mRNA levels of HIF-1α target genes, CA9, GLUT1, HK1 and VEGFA1, consistent with destabilization of the transcription factor at 100 ppm H_2_S (Fig. 2*E*). Since acute and chronic H_2_S similarly destabilized HIF-1α, we used these regimes interchangeably, depending on whether a relatively fast response (*e.g*., inhibition of new protein synthesis) or a slower response (*e.g*., sensor fluorescence) was being monitored.

### H_2_S shifts the threshold for hypoxia sensing

We expressed the genetically encoded fluorescent hypoxia sensors, UnaG and dUnaG, which are controlled by HIF-1α-dependent recognition of the hypoxia-responsive element (HRE) (Fig. 2*F*) (33). UnaG- and dUnaG-expressing HT-29 cells grown at 2% O_2_ exhibited robust fluorescence compared with empty vector (EV) controls (Figs. 2*G*, S3*A*). In the presence of increasing H_2_S, the decrease in fluorescence paralleled HIF-1α destabilization (Figs. 2*H*, S3*D*). The sensors were also tested in HEK293 cells, which showed a qualitatively similar response to H_2_S (Fig. S3, *B*, *C*, *E* and *F*). The possibility that H_2_S directly quenches fluorescence was ruled out by treating UnaG-expressing HEK293 cells with a large bolus of H_2_S (1 mM), which had no effect on hypoxia-induced fluorescence (Fig. S3, *G* and *H*).

As expected, UnaG and dUnaG fluorescence in HT-29 and HEK293 cells decreased as the O_2_ concentration was increased from 2% to 21% (Figs. 2, *I* and *J*, S4, *A*–*F*). The effective intracellular O_2_ sensed in cells grown at 2% O_2_ in the presence of H_2_S could then be estimated from the O_2_ dependency curves of the fluorescent sensors. Even at low concentrations, H_2_S profoundly affected the HIF-1α response and HT-29 cells grown in 2% O_2_. Thus, in the presence of 25 or 100 ppm H_2_S, UnaG and dUnaG reported the equivalent 5% or 15% O_2_, respectively (Figs. 2*J*, S4*D*). In contrast, the sensor response at 2% O_2_ ± 10 ppm H_2_S was indistinguishable, indicating that the cellular capacity for sulfide oxidation protected against O_2_ accumulation at this concentration of H_2_S and/or resulted from the limited sensitivity of the sensor to small changes in O_2_ levels. Similar estimates for an H_2_S-dependent increase in effective O_2_ were obtained with HEK293 cells, which reported the equivalent of 9% or ∼14% O_2_ with 25 or 100 ppm H_2_S, respectively (Fig. S4, *E* and *F*, Table S2).

### SQOR levels correlate inversely with HIF-1α sensitivity to H_2_S

**Figure 3:**
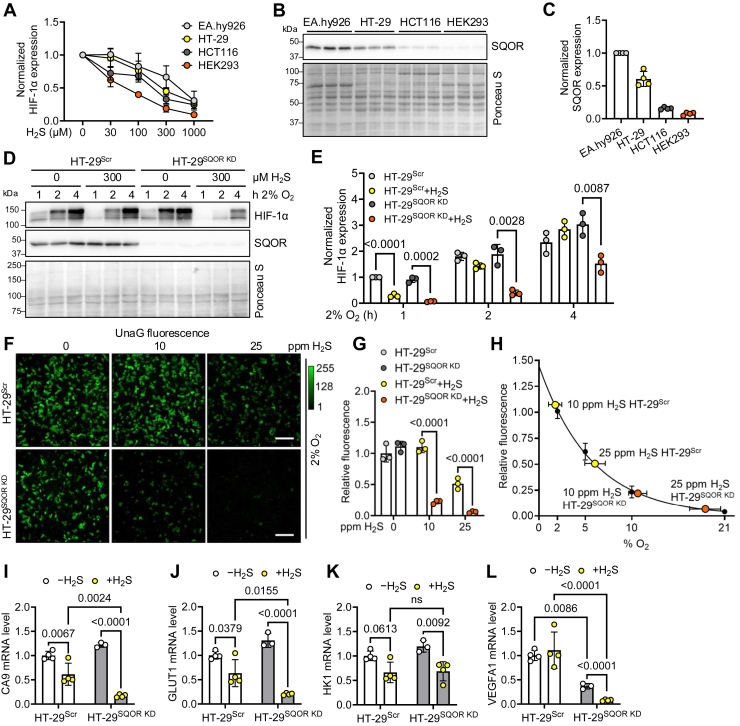
**SQOR expression affects HIF-1α sensitivity to H_2_S.***A*, HIF-1α levels relative to untreated controls in EA.hy926, HT-29, HCT116, and HEK293 cells grown at 2% O_2_ and varying Na_2_S (0–1 mM). *B*, SQOR expression in EA.hy926, HT-29, HCT116, and HEK293 cell lysates detected by Western blotting. *C*, quantitation of SQOR levels in *B* normalized to Ponceau S staining relative to levels in EA.hy926 cells, which have the highest expression (n = 4). *D*, HIF-1α expression in HT-29^Scr^*versus* HT-29^SQOR KD^ cells in 2% O_2_ treated with 300 μM H_2_S for 1 to 4 h. *E*, quantitation of HIF-1α levels in *D* normalized to Ponceau S staining and shown relative to levels in HT-29^Scr^ cells after 1 h (n = 3). *F*, effect of H_2_S (10 and 25 ppm, 24 h) on UnaG expression in HT-29^Scr^ and HT-29^SQOR KD^ cells grown in 2% O_2_. The images are representative of three independent experiments. The scale bar represents 200 μm. *G*, quantitation of data in *F* normalized to HT-29^Scr^ without H_2_S. *H*, UnaG fluorescence in *G* was plotted on the standard curve for UnaG fluorescence *versus* O_2_ concentration. *I*–*L*, expression of HIF target genes in HT-29^Scr^ and HT-29^SQOR KD^ cells grown in 2% O_2_ ± 25 ppm H_2_S for 24 h. Expression of (*I*) CA9, (*J*) GLUT1, (*K*) HK1, and (*L*) VEGFA1 mRNA normalized to the mean of three reference genes (n = 3 or 4). A two-way ANOVA followed by Šídák post hoc test was performed for statistical analysis; the data represent the mean ± SD. HEK293, human embryonic kidney 293 cell line; HIF, hypoxia-inducible factor; H_2_S, hydrogen sulfide; SQOR, sulfide quinone oxidoreductase.

Comparison of HIF-1α destabilization across four cell lines revealed different sensitivities to H_2_S (Fig. 3*A*) and raised the question as to whether the differences were correlated with their sulfide oxidation capacity. We found an inverse correlation between SQOR expression levels and HIF-1α destabilization (Fig. 3, *B* and *C*), consistent with a primary role for this enzyme in regulating intracellular H_2_S, and, therefore, O_2_ levels (Fig. 1*A*). Accordingly, the H_2_S effect was exacerbated when SQOR was knocked down (in HT-29^SQOR KD^), which were slower to regain stable HIF-1α expression compared with scrambled controls (HT-29^Scr^) (Fig. 3, *D* and *E*).

UnaG- and dUnaG-expressing HT-29^SQOR KD^ cells were hypersensitive to low (10 ppm) and moderate (25 ppm) H_2_S exposure, resulting in ∼80% and >95% decrease in fluorescence intensity, respectively, compared with control cells (Figs. 3, *F* and *G*; S5, *A* and *B*). The fluorescence corresponded to intracellular O_2_ levels of 10% to 14% and 18% to 23% for 10 and 25 ppm H_2_S, respectively (Figs. 3*H*, S5*C*, Table S2). Furthermore, HT-29^SQOR KD^ cells exhibited greater HIF-1α destabilization than scrambled controls when grown at moderate H_2_S (25 ppm, 24 h) (Fig. S5, *D* and *E*). These results were mirrored by the suppression of HIF-1α target genes with the exception of HK1 (Fig. 3, *I*–*L*).

### H_2_S activates PHD to destabilize HIF-1α

**Figure 4:**
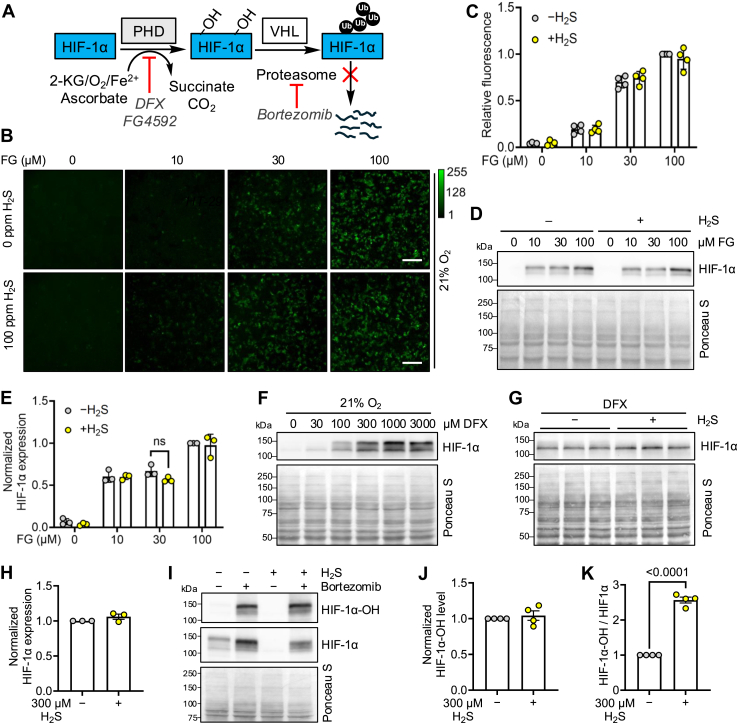
**Increased PHD activity mediates hypoxic HIF destabilization by H_2_S.***A*, scheme showing targets of select inhibitors of the O_2_-dependent HIF degradation pathway. *B*, UnaG-expressing HT-29 cells were cultured for 24 h in 21% O_2_ ± 100 ppm H_2_S in the presence of 0, 10, 30, or 100 μM FG4592 (FG). The scale bar represents 200 μm. *C*, quantitation of data in *B* expressed relative to the 100 μM FG minus H_2_S condition (n = 4). *D*, HIF-1α expression in HT-29 cells treated with 0, 10, 30, and 100 μM FG and ± 100 ppm H_2_S. *E*, quantitation of data in *D* normalized to Ponceau S staining (n = 3). *F*, Western blot analysis of HIF-1α in HT-29 cells grown in 21% O_2_ and treated with varying concentrations of the PHD inhibitor deferoxamine (DFX). *G*, HIF-1α expression in HT-29 cells grown in 21% O_2_ ± 300 μM DFX and 300 μM Na_2_S for 1 h. *H*, quantitation of data in *G* normalized to Ponceau S staining (n = 3). *I*, HT-29 cells were pretreated with bortezomib for 6 h and then grown in 2% O_2_ ± 300 μM Na_2_S. The hydroxylated form of HIF-1α was detected by Western blot analysis. *J* and *K*, quantitation of data in *I* normalized to Ponceau S staining (*J*) or total HIF-1α (*K*) (n = 4). Data represent the mean ± SD. Statistical analysis was performed with a one-way ANOVA followed by Tukey's post hoc test (*C*, *E*) or an unpaired *t* test (*H*, *J*, and *K*). HIF, hypoxia-inducible factor; H_2_S, hydrogen sulfide; KG, α-ketoglutarate; PHD, prolyl hydroxylase.

The effect of H_2_S on the destabilization of HIF-1α could be exerted at or downstream of PHD, which requires 2-ketoglutarate and iron in addition to O_2_ for activity (Fig. 4*A*). The PHD inhibitor, roxadustat or FG4592, induced a dose-dependent (0–100 μM) increase in UnaG fluorescence in cells grown at 21% O_2_, which was insensitive to H_2_S (Figs. S6*A*; 4, *B* and *C*). HIF-1α stability was unaffected by acute or chronic H_2_S treatment in the presence of FG4592 (Figs. 4, *D* and *E*, S6, *B* and *C*). H_2_S similarly had no effect on HIF-1α stability in the presence of the iron chelator deferoxamine (DFX) (Fig. 4, *F*–*H*). These results are consistent with the model that PHD activity is required for H_2_S-dependent HIF-1α destabilization. In the presence of H_2_S and the proteasome inhibitor bortezomib, HIF-1α levels declined, but an increase in the proportion of hydroxylated HIF-1α relative to the total HIF-1α pool was seen (Fig. 4, *I*–*K*). In bortezomib-treated cells, a high molecular weight smear of HIF-1α was observed that we ascribe to the ubiquitinated species, which increased relative to total HIF-1α in H_2_S-treated cells (Fig. S6, *D*–*F*). Together, these data indicate that H_2_S increases PHD activity under hypoxia.

In principle, H_2_S could additionally destabilize HIF-1α levels by inhibiting new protein synthesis (34) (Fig. S7*A*). This mechanism was, however, ruled out since the effect of puromycin, which allows monitoring of nascent polypeptide synthesis, was unaffected by acute or chronic H_2_S (Fig. S7, *B*–*D*). Collectively, these results support a model in which H_2_S stimulates substrate (*i.e.*, O_2_)-level activation of PHD to enhance HIF degradation. Furthermore, eukaryotic initiation factor 2α (eIF2α) phosphorylation was unaffected by sulfide (Fig. S7, *E*–*H*), as reported previously (21), which is in contrast to H_2_S-dependent enhancement of phospho-eIF2α in mouse embryonic fibroblast and HeLa cells (35). It is unclear whether this disparity reflects differences in cell lines and/or stem from other mechanistic differences such as H_2_S-dependent inhibition of protein phosphate PP1c in some but not other cells.

### *Lactobacillus brevis* NADH oxidase counters the effect of H_2_S on HIF-1α

**Figure 5:**
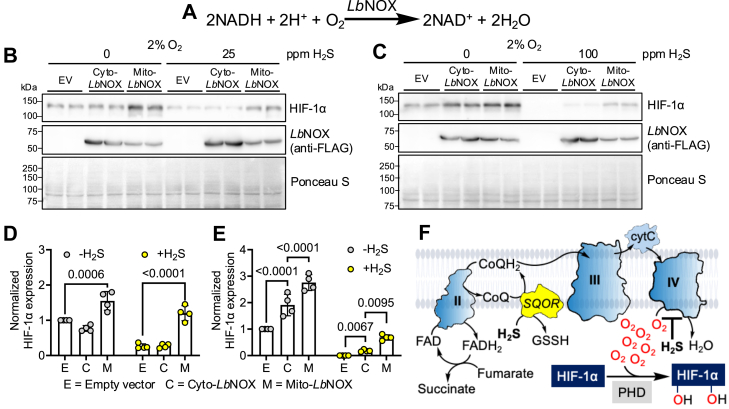
***Lb*NOX restores HIF under H_2_S.***A*, O_2_ consumption reaction catalyzed by *Lb*NOX. *B* and *C*, Western blot analysis of HIF-1α and *Lb*NOX expression in HT-29 cells transfected with empty vector (EV), cytosolic *Lb*NOX (Cyto-*Lb*NOX), or mitochondrial *Lb*NOX (Mito-*Lb*NOX) and cultured in 2% O_2_ ± 25 ppm H_2_S (*B*), or ± 100 ppm H_2_S (*C*) for 24 h. *D* and *E*, quantitation of HIF-1α data in *B* (*D*) and *C* (*E*) normalized to Ponceau S staining (n = 4). *F*, O_2_ and fumarate serve as terminal electron acceptors for H_2_S oxidation, allowing sulfide concentration to dynamically regulate intracellular O_2_ levels. Data represent mean ± SD. Statistical analysis (*D* and *E*) was performed with a two-way ANOVA followed by Tukey's post hoc test; HIF, hypoxia-inducible factor; H_2_S, hydrogen sulfide; *Lb*NOX, *Lactobacillus brevis* NADH oxidase.

As a further test of our hypothesis that H_2_S destabilizes HIF-1α by increasing intracellular O_2_ levels, we used *Lactobacillus brevis* NADH oxidase (*Lb*NOX), to promote O_2_ consumption (36) by dissipating the NADH pool (Fig. 5*A*). Mitochondrial but not cytoplasmic expression of *Lb*NOX moderately attenuated HIF-1α destabilization induced by a low concentration of sulfide (25 ppm, 24 h in 2% O_2_) (Fig. 5, *B* and *D*). When the sulfide concentration was raised to 100 ppm, inducing complete suppression of HIF-1α, cytoplasmic and mitochondrial *Lb*NOX partially reversed HIF destabilization, with mitochondrial *Lb*NOX having a greater effect (Fig. 5, *C* and *E*).

## Discussion

The cartography of intracellular O_2_ is dynamically regulated by mitochondrial respiration and is important for protecting against O_2_ cytotoxicity (37, 38). Consequently, mitochondrial diseases are characterized by dysregulated O_2_ metabolism and venous hyperoxia during exercise (39), and interestingly, show promise for hypoxia therapy (40). In this study, we establish H_2_S as a powerful regulator of intracellular O_2_, with important implications for O_2_ sensing and redox metabolism, especially in tissues with low physiological O_2_.

Using a custom sulfide growth chamber to simulate chronic H_2_S exposure (32, 41), we demonstrated that even a low concentration of H_2_S (25 ppm in the atmosphere or 4 μM in culture medium) induces a profound shift such that 2% O_2_ elicits a response equivalent to 5% to 9% ambient O_2_ (Fig. 2*J* and Table S2). H_2_S exhibits a dose-dependent modulation of O_2_ tension that is sensitive to SQOR levels across cell lines (Fig. 3, *A*–*C*) and is consistent with its importance in a high turnover rate for intracellular H_2_S (42, 43). We postulate that SQOR activity in response to H_2_S biogenesis or exogenous exposure from the gut lumen regulates ETC flux, and therefore, O_2_ metabolism in healthy tissue (44, 45). Furthermore, we have recently demonstrated that H_2_S autoactivates SQOR by increasing the proportion of the active trisulfide–containing form of the enzyme (46). The physiological significance of these observations is underscored by the importance of SQOR for endothelial cell proliferation under hypoxic conditions, postischemia neovascularization, tumor neoangiogenesis, and rescue from hypoxic brain injury (47, 48).

SQOR is ubiquitously expressed, and based on the Human Protein Atlas database, is present at highest levels in the gastrointestinal tract, skeletal muscle, nasopharynx, and kidney. Whole-body SQOR-deficient mice cease to grow at weaning and die within 8 to 10 weeks of age (48, 49), indicating severe growth impairment in the absence of H_2_S oxidation capacity. Inherited SQOR deficiency presents as Leigh syndrome (50), a disease with over 75 contributing genetic loci, which is characterized by profound neurological and neuromuscular defects (51). In contrast to murine models, the clinical presentation in the limited number of SQOR-deficient patients described so far has ranged from 4 to 8 years of age, possibly suggesting incomplete penetrance of the associated variants (50).

H_2_S-dependent regulation of O_2_ consumption and accumulation is paradoxical since the latter comes at the cost of inhibiting respiration and decreasing O_2_-dependent sulfide clearance (5, 45). However, sulfide consumption and O_2_ accumulation can occur simultaneously because of the flexibility inherent in the ETC for coupling H_2_S oxidation to fumarate *versus* O_2_ reduction (52, 53) (Fig. 5*F*). We posit that physiologically relevant hypoxia modulation can occur within an H_2_S concentration window where complex IV is partially inhibited and contributes to regional O_2_ contours that can vary significantly even within a given tissue (18, 54). The durability of the complex IV response to fractional inhibition, even at low H_2_S (55), further suggests that the effect of sulfide on O_2_ accumulation might be long lived. The physiological importance of sulfide-dependent O_2_ regulation is revealed by the loss of Fe–S proteins in the colon of mice lacking SQOR in intestinal epithelial cells (Fig. 1, *F* and *G*). We presume that damage accrues at O_2_ and ROS-sensitive sites as H_2_S, and therefore O_2_, increases. We speculate that the beneficial boost in exercise endurance by the natural product ergothioneine, which increases endogenous H_2_S biogenesis (56, 57), could be due in part to increased O_2_ availability across skeletal muscle.

Mitochondria function as O_2_ sinks (58) and help establish intracellular O_2_ gradients (59, 60, 61). Perinuclear mitochondria decrease nuclear O_2_ exposure and limit its genotoxicity (62). The effectiveness of mitochondrial, but not cytoplasmic, *Lb*NOX to rescue hypoxia sensing was dependent on H_2_S concentration, further illuminating the interplay between O_2_ and H_2_S and its sensitivity to their respective concentrations. Decreased ETC flux because of H_2_S leads to a reductive shift in the NAD pool (26), which can be dissipated by *Lb*NOX that concomitantly utilizes O_2_ (Fig. 5*A*). Since HIF is a cytoplasmic protein, it is curious that mitochondrially targeted *Lb*NOX was more effective in stabilizing HIF-1α, which could be explained in part by its higher O_2_ consumption (36). In addition, by increasing coenzyme Q availability, mito-*Lb*NOX activity could enhance SQOR-dependent H_2_S clearance, decreasing complex IV inhibition. Importantly, in addition to PHD (*K*_*M*(O2)_ = 67–85 μM (63)), other enzymes that exhibit varying degrees of unsaturation at ambient intracellular O_2_ (64) are also expected to be sensitive to H_2_S-mediated changes in O_2_ (5). Some notable examples include the KDM family of lysine demethylases that use histone and nonhistone substrates and the TET enzymes that catalyze hydroxylation of 5-methyl cytosine on DNA. These functions are central to epigenetic regulation and other processes such as mitochondrial biogenesis (64).

In summary, we demonstrate that H_2_S shifts the threshold for hypoxia sensing across cell lines by increasing intracellular O_2_. Chronic H_2_S exposure under ambient hypoxia induces an oxidative shift in the mitochondrial cysteine proteome and destabilizes Fe–S proteins involved in oxidative energy metabolism in cells. In a physiological setting, the loss of SQOR in gut epithelium renders the tissue vulnerable to oxidative changes and reveals that sulfide oxidation capacity buffers O_2_ cytotoxicity (5). Perturbed O_2_ metabolism is likely to underlie the pathologies associated with H_2_S overload in SQOR (50), ETHE1 (65), or sulfite oxidase (66) deficiency, and our study provides a foundation for investigating these links in these and other diseases, including acute respiratory distress syndrome and inflammation that are characterized by hyperoxia. Finally, increasing H_2_S synthesis or decreasing its oxidation might be beneficial in diseases characterized by hypoxia, including metabolic and cardiovascular diseases.

### Limitations of our study

While our study provides a quantitative evaluation of the magnitude of H_2_S-induced perturbation of the intracellular O_2_ economy in the setting of 2% to 10% ambient O_2_, the limited dynamic range of the UnaG and dUnaG hypoxia sensors restricted their utility outside this O_2_ concentration range. Second, while the *in vivo* mouse colon data are consistent with H_2_S-dependent dysregulation of O_2_ metabolism, our experiments were largely focused on cell lines adapted to long-term culture under normoxic conditions. Finally, our study assessed the effect of exogenous H_2_S at low O_2_ to simulate the environment in the colon lumen. Endogenous upregulation of H_2_S biogenesis *via* diet (*e.g.*, increased methionine and/or cysteine) and/or *via* overexpression of the H_2_S-synthesizing enzymes would broaden the scope of our findings and uncover its regulation in other contexts.

## Experimental procedures

### Materials

RPMI1640 medium (11875093), RPMI + Hepes (22400089), Dulbecco's modified Eagle's medium (11995065), fetal bovine serum (A5256701), 1x penicillin–streptomycin (Pen–Strep; 15140122), geneticin (10131035), trypsin–EDTA (25300062), and PBS (10010023) were from Gibco. Sodium sulfide nonahydrate (431648), dimethyl sulfoxide (D2650), protease inhibitor (P8340), doxycycline (D3447), and puromycin (P8833) were from Sigma–Millipore. Methanol (A4544) and Tween-20 (BP337500) were from Fisher. Polyvinylidene difluoride membranes (1620177), blot filter paper (1703932), and Clarity Western Enhanced Chemiluminescence Substrate (102032117 and 102032118) were from Bio-Rad. DFX (14595), bortezomib (10008822), and FG4592 (15294) were from Cayman. Phosphatase inhibitor cocktail (HY-K0021) was from MedChemExpress. Nonidet P40 (74385) was from Fluka BioChemika. Gas cylinders of H_2_S (500 l 5000 ppm H_2_S in N_2_ within 1% accuracy), normal air containing CO_2_ (21% O_2_, 5% CO_2_, and 74% N_2_), and hypoxic air (2% O_2_, 5% CO_2_, and 93% N_2_) were from cryogenic gases (Detroit).

The primary antibodies used in this study were anti-HIF-1α (Abcam, ab179483, 1:3000 dilution), anti-HIF-2α (R&D Systems, AF2997, 1:1000 dilution), anti-HIF-1α-OH Pro-564 (CST, 3434, 1:3000 dilution), anti-MT-CO2 (Abcam, ab110258, 1:5000 dilution), anti-SQOR (Proteintech, 17256-1-AP, 1:5000 dilution), antipuromycin (DSHB PMY-4A4, 1:500 dilution), anti-eIF2α (CST, 9722, 1:3000 dilution), anti-p-eIF2α phospho-S51 (Abcam ab32157, 1:3000 dilution), anti-NDUFS8 (Abcam, ab170936, 1:1000 dilution), anti-ACO2 (Abcam, ab129069, 1:10,000 dilution), anti-LIAS (Abcam, ab246917, 1:1000 dilution), anti-UQCRSF1 (Abcam, ab14746, 1:1000 dilution), anti-FLAG (Sigma, F1804, 1:5000 dilution). Secondary antibodies conjugated to horseradish peroxidase were anti-rabbit (Abcam, ab6721) and anti-mouse (Abcam, ab205719) used at a 1:10,000 dilution.

### Cell culture

HT-29, HCT116, HEK293, and EA.hy296 cells were obtained from the American Type Culture Collection. HT-29^Scr^, HT-29^SQOR^ ^KD^, HT-29 cyto-*Lb*NOX, HT-29 mito-*Lb*NOX, and HT-29 EV cells were previously generated in the laboratory (26, 31). Cell lines were tested in-house and were found to be free of mycoplasma contamination. HT-29 HRE-UnaG and HT-29 HRE-dUnaG stable cells were generated in this study as described below. HT-29 cells were cultured in RPMI1640 medium supplemented with 10% fetal bovine serum (FBS) and 1x Pen–Strep. All other cell lines were cultured in Dulbecco's modified Eagle's medium with 10% FBS and 1x Pen–Strep. HT-29 cells stably expressing cyto-*Lb*NOX, mito*Lb*NOX, EV, HRE-UnaG, and HRE-dUnaG were supplemented with 300 μg/ml geneticin. HT-29^SQOR KD^ and HT-29^Scr^ were supplemented with 1 μg/ml puromycin. All cell lines were cultured in a humidified cell culture incubator at 37 °C with 5% CO_2_ and ambient air, except in hypoxia experiments, which were conducted at 2% O_2_, 93% N_2_, and 5% CO_2_, unless otherwise noted. During 24 h hypoxia experiments, cells were switched to fresh medium containing Hepes at twice the normal volume to minimize acidification (32).

### Generation of stable cell lines for HRE-UnaG and HRE-dUnaG

Plasmids for expression of the UnaG and dUnaG fluorescent proteins under control of an HRE promoter were obtained from Addgene (plasmids #201710 and 201711). Transfection-grade plasmids were purified from *Escherichia coli* (Qiagen, 12123), and the insert region was confirmed by sequencing (Eurofins). HT-29, HT-29^Scr^, and HT-29^SQOR KD^ cells were transfected with the HRE-UnaG and HRE-dUnaG expression plasmids in 24-well culture plates. After 24 h, cells were switched to fresh medium containing geneticin at 300 μg/ml. Cells were incubated in the presence of geneticin for 2 to 3 weeks, after which all nontransfected cells had died. Surviving cells were expanded in 6-well plates, and a portion of the cells were split evenly into two 24-well plates for a trial experiment. One plate was left in normal cell culture conditions, and the other was incubated under hypoxia for 24 h. The fluorescence in each well was quantitated using an EVOS microscope (EVOS M5000), and the colonies with the highest fluorescence for each plasmid were expanded to make cell stocks.

### Western blotting

#### Sample preparation

Cells were seeded 24 h prior to the start of experiments in 35-mm or 60-mm plates such that the confluency at collection was 70% to 80%. Fresh medium was added at the start of the experiments. For detection of protein expression, cell plates were washed with ice-cold PBS and then scraped with a cell scraper on ice in lysis buffer (20 mM Hepes [pH 7.4], 25 mM KCl, 0.5% Nonidet P40, and 1x protease inhibitor) and immediately frozen on dry ice. Samples were stored at −80 °C until further use.

#### Development of SDS-PAGE Western blots

Frozen lysates were rapidly thawed in a 37 °C water bath for 50 s until the ice was nearly melted and then vortexed for 15 s and immediately centrifuged at 13,000*g* for 5 min at 4 °C. Cleared lysates were transferred to new tubes and immediately denatured with 4x SDS + DTT buffer (200 mM Tris–HCl [pH 6.8], 40% glycerol, 8% SDS, 0.1% bromophenol blue, and 400 mM DTT) and incubated for 5 min at 80 °C. In parallel, 4 μl cleared lysate per sample was used for protein assay with 1 ml Bradford reagent (Bio-Rad). Samples (30 μg per lane) were run on 8%, 10%, or 12% Tris–glycine SDS gels at 100 to 130 V, transferred to polyvinylidene difluoride membranes, and blocked for 30 min with 5% milk in Tris-buffered saline containing 0.1% Tween-20. Membranes were probed overnight with primary antibodies diluted in 5% milk and then washed 3 × 10 min with Tris-buffered saline containing 0.1% Tween-20. Horseradish peroxidase–linked secondary antibodies were incubated for 1 to 2 h with the membranes, followed by another round of 3 × 10 min washes. Blots were developed with enhanced chemiluminescence substrate and imaged with a Bio-Rad ChemiDoc imaging system. Equal loading was verified by Ponceau S staining of membranes. Blot image files were quantitated by pixel intensity in ImageJ and normalized to the quantified Ponceau S staining. When two bands were seen with the HIF-1α antibody, the intensities of both were combined for quantitation.

### Intracellular O_2_ assay

HEK293 cells were plated at 50% confluency in 6-well plates. After 24 h to allow attachment to the plate, cells were transfected with HRE-UnaG, HRE-dUnaG, or EV with Lipofectamine 3000 (Invitrogen, L3000001). After 24 h of transfection, cells were split evenly between three 24-well plates. After another 24 h to allow reattachment of cells, 1 ml fresh medium was carefully added to each well, and the plates were incubated in the following atmospheres: 1) a baseline hypoxia control (2% O_2_, 5% CO_2_, and 93% N_2_); 2) hypoxia as in 1) admixed with 10, 25, or 100 ppm H_2_S as described previously (32); and 3) a calibration control at either 5%, 10%, or 21% O_2_, 5% CO_2_, with the balance being N_2_. After 24 h of incubation, all plates were imaged using the EVOS microscope at 20x magnification using GFP filter and brightfield to obtain representative images. Images were quantitated using ImageJ. A calibration bar denoting the pixel intensity range is provided in each figure. For HT-29 cells stably expressing HRE-UnaG and HRE-dUnaG, a similar procedure was followed, except that the transient transfection steps were omitted. Instead, cells were plated directly in 3 × 24-well plates at ∼40% confluency 24 h prior to switching to hypoxia.

### HIF stability assays

#### Bolus H_2_S treatment

HT-29, HEK293, HCT116, and EA.hy296 cells were plated in 6 cm dishes and grown to 70% to 80% confluency. On the day of the experiment, fresh medium supplemented with a bolus of Na_2_S was added, and cells were immediately transferred to the hypoxia chamber. HIF was detected from the cell lysates and normalized to Ponceau S staining.

#### H_2_S time-course and dose–response assays

For the time-course experiments, HT-29 cells (70–80% confluency) were cultured in 6 cm plates with fresh growth medium and treated with either vehicle or 300 μM Na_2_S and then immediately placed in the hypoxia chamber. Plates were removed from the chamber for lysate collection at intervals ranging from 0.25 to 4 h. For dose–response experiments, cells treated with varying doses of Na_2_S (0–1000 μM) were incubated under 2% hypoxia for 1 h. HIF was detected in the cell lysates, and blots were normalized by Ponceau S staining. Na_2_S-treated cells were cultured separately from non–Na_2_S-treated cells to prevent contamination of aerosolized H_2_S in the culture medium of the control samples.

#### PHD inhibitor treatments

HT-29 cells were cultured under normoxic conditions for 1 h in medium treated with 0, 30, 100, 300, 1000, or 3000 μM DFX or with 0, 3, 10, 30, 100, or 300 μM FG4592 dissolved in 0.1% dimethyl sulfoxide (DMSO). HIF was detected in cell lysates and normalized to Ponceau S staining.

#### HIF ubiquitination and proline hydroxylation assays

HT-29 cells were pretreated with the proteasome inhibitor bortezomib (1 μM for 6 h) and then with a bolus of Na_2_S and incubated under hypoxia for 1 h. Cell lysates were isolated, run on 8% gels, and probed with anti–HIF-1α to observe high molecular weight bands corresponding to ubiquitinated HIF. Blots were probed with anti-HIF-1α-OH to detect the proline hydroxylated form. Ubiquitinated or hydroxylated HIF-1α were normalized to total HIF-1α levels and Ponceau S staining.

#### LbNOX assays

HT-29 cells expressing *Lb*NOX and mito-*Lb*NOX (31) were plated in 6-cm plates, and *Lb*NOX expression was induced with 300 ng/ml doxycycline for 24 h. Cells were moved to the hypoxia chamber and exposed to either 0, 25, or 100 ppm H_2_S for 24 h and then harvested for protein immunoblotting.

### Puromycin incorporation assay

Cells were switched to medium containing 1 μM puromycin and coincubated for 1 h with 300 μM Na_2_S. Cell lysates were probed with antipuromycin antibody to detect puromycin-conjugated nascent peptides as a marker of new protein synthesis. A negative control was run without puromycin. Western blots were normalized to Ponceau S staining.

### RT–qPCR analysis

RNA was extracted from 70% to 80% confluent cells using the Trizol reagent following the manufacturer’s protocol and reverse transcribed with murine leukemia virus using random primers. Samples were analyzed by qPCR using SYBR Select Master Mix, and the primers are listed in Table S3. All data were normalized to the mean of three reference genes, *GUSB*, *TBP*, and *POLR2A*.

### Lipid peroxide assay

All fluorescence-activated cell sorting analyses were conducted using the Bio-Rad Ze5 multilaser, high-speed cell analyzer operated with the Everest software package at the University of Michigan Flow Cytometry Core Facility. All data were analyzed using FlowJo (version 10.8.1, Becton, Dickinson and Company).

### Lipid ROS analyses using the BODIPY lipid peroxide sensor

HT29 cells were split at two million cells per 6 cm plate, cultured overnight, and then had their medium changed to 8 ml before plating for 24 h culture ± 100 ppm H_2_S. Two hours before harvest, positive control cells were treated with 100 μM cumyl hydroperoxide, which was prepared fresh in absolute ethanol as a 1:100 dilution of a 100 mM, 80% technical grade stock (Fisher, AAL0686622). A vehicle control of 10 μl absolute ethanol was included. After 24 h ± 100 ppm H_2_S, the cells were washed twice with 1 ml ice-cold PBS, trypsin digested, pelleted at 1000*g* for 5 min at 4 °C, and then resuspended in 3 ml of ice-cold blocking buffer (10% FBS in PBS). The cells were transferred to 2 × 1.5 ml tubes. Each tube was centrifuged at 1000*g* for 5 min and then resuspended in prewarmed blocking buffer containing DMSO vehicle or 2 μM of BODIPY and allowed to stain in the dark for 30 min at 37 °C. The DMSO unstained samples were not included in the results but were used to confirm high signal to noise. After staining, the cells were centrifuged at 1000*g* for 5 min, resuspended in 2% FBS in PBS, and then filtered through 5-mL BD round-bottom falcon tubes with cell-strainer caps for fluorescence-activated cell sorting analysis on the Bio-Rad Ze5 multilaser. FlowJo (version 10.8.1) was used to conduct gating and ratio the PE and FITC fluorescence emission spectra for each analyzed cell, to plot the histograms of the results, and to calculate the median fluorescence ratios.

### Proximity labeling oxidative isotope–coded affinity tag analysis of the cysteine redox proteome

HEK293T cells expressing PDK1-TurboID were split at 10% confluency into 10 cm plates and cultured for 2 days before changing medium and growing with or without 100 ppm H_2_S for an additional 24 h. Then, each plate was treated with 500 μM biotin in DMSO for 1 h before washing 3x with cold Dulbecco's PBS. Cells were harvested by scraping in Dulbecco's PBS, pelleting at 1600*g* for 5 min, aspirating the supernatant, and frozen in liquid nitrogen. Cells were 90% to 95% confluent at the time of harvest. Frozen cells were then subjected to the redox proteomics workflow as described (28).

### Statistics

All data are presented as mean ± SD with the individual data points displayed. For Western blot and fluorescence quantitation, control group data were set to 1, and other data points were displayed as relative levels compared with the controls. Statistical analyses were performed with GraphPad Prism 10.2.2 (GraphPad Software, LLC). Unpaired, two-tailed *t* tests were used for binary comparisons. One- or two-way ANOVA followed by either Šídák's or Tukey's post hoc test was used for multiple comparisons. The exact *p* values are displayed above each indicated comparison. Not significant (ns) indicates a *p* value >0.05.

## Data availability

All data generated and analyzed in this study are included in the main text and supporting information file.

## Supporting information

This article contains supporting information.

## Conflict of interest

R. B. is a consultant for Zyphore Therapeutics and Alnylum Pharmaceuticals.
