# Supplementary material for: Hydrogen sulfide increases intracellular oxygen and inhibits the HIF response
Source: J Biol Chem. 2026 Jan 12;302(3):111151. doi: 10.1016/j.jbc.2026.111151 (PMC12925153; doi:10.1016/j.jbc.2026.111151)
Supplement: Supporting information [file mmc1.docx]

Supplementary Information

**Hydrogen sulfide increases intracellular oxygen and inhibits the HIF response**

Joseph Brake^1^, David A Hanna^1^, Roshan Kumar^1^, Qianni Peng^2^, Aaron P. Landry^1^, Rashi Singhal^3^, Eranthie Weerapana^2^, Yatrik M. Shah^3,4,5^ and Ruma Banerjee^1^

Departments of ^1^Biological Chemistry, University of Michigan, Ann Arbor, MI 48109; ^2^Chemistry Department, Boston College, Chestnut Hill, MA 02467; ^3^Molecular and Integrative Physiology, ^4^Internal Medicine, ^5^Rogel Cancer Center, University of Michigan, Ann Arbor, MI 48109

*Corresponding author email: rbanerje@umich.edu

**Supplementary Table of Content**

**Supplementary Table 1.** Mass spectrometric data from PL-OxICAT analysis

**Supplementary Table 2**. Perturbation of intracellular O_2_ levels by H_2_S

**Supplementary Table 3.** List of primers used for RT-qPCR analysis

**Supplementary Figure 1**. H_2_S increases lipid oxidation

**Supplementary Figure 2**. HIF destabilization by H_2_S across cell lines

**Supplementary Figure 3**. H_2_S-induced increase in O_2_ monitored by dUnaG

**Supplementary Figure 4.** O_2_ calibration curves in HT-29 and HEK cells

**Supplementary Figure 5:** SQOR deficiency sensitizes cells to HIF destabilization by H_2_S

**Supplementary Figure 6.** HIF destabilization by H_2_S is PHD-dependent

**Supplementary Figure 7.** H_2_S does not affect HIF-1α synthesis

**Supplementary Table 2**. Perturbation of intracellular O_2_ levels by H_2_S in HT-29 and HEK cells grown in 2% O_2_ and exposed to the indicated concentrations of sulfide.

| Cell Line | [H_2_S], ppm | % O_2_, Intracellular^1^ | |
| --- | --- | --- | --- |
|  |  | UnaG | dUnaG |
| HT-29 | 10 | 2.0 ± 0.4 | 1.9 ± 0.2 |
| HT-29 | 25 | 5.2 ± 0.2 | 6.3 ± 1.1 |
| HT-29 | 100 | 15.1 ± 3.5 | 17.2 ± 1.1 |
| HEK293 | 10 | 2.0 ± 0.1 | 2.0 ± 0.2 |
| HEK293 | 25 | 8.5 ± 1.4 | 9.5 ± 1.3 |
| HEK293 | 100 | 14.2 ± 4.8 | 13.5 ± 5.2 |
| HT-29^Scr^ | 10 | 1.6 ± 0.5 | 1.9 ± 0.7 |
| HT-29^Scr^ | 25 | 5.9 ± 1.0 | 5.2 ± 0.8 |
| HT-29^SQOR KD^ | 10 | 10.8 ± 0.8 | 14.0 ± 0.6 |
| HT-29^SQOR KD^ | 25 | 18.3 ± 1.6 | 23.6 ± 1.7 |

^1^O_2_ levels were estimated relative to 2% ambient O_2_ and 0 ppm H_2_S

**Supplementary Table 3**. List of primers used for RT-qPCR analysis.

| Gene | Sequence (5’ 🡪 3’) |
| --- | --- |
| Ca9 | F: GTTTCCCTGCCGAGATCCAC |
|  | R: AGAGGGCAGGAGTGCAGATA |
| HK1 | F: AGCACGTTTGCACCATTGTC |
|  | R: AAACGCCGGGAATACTGTGG |
| SLC2A1 (GLUT1) | F: CTTCACTGTCGTGTCGCTGT |
|  | R: GGCCACGATGCTCAGATAGG |
| VEGFA1 | F: ACAACAAATGTGAATGCAGACCA |
|  | R: GAGGCTCCAGGGCATTAGAC |
| GUSB | F: CTGTCACCAAGAGCCAGTTCCT |
|  | R: GGTTGAAGTCCTTCACCAGCAG |
| POLR2A | F: GAGAGCGTTGAGTTCCAGAACC |
|  | R: TGGATGTGTGCGTTGCTCAGCA |
| TBP | F: TGTATCCACAGTGAATCTTGGTTG |
|  | R: GGTTCGTGGCTCTCTTATCCTC |


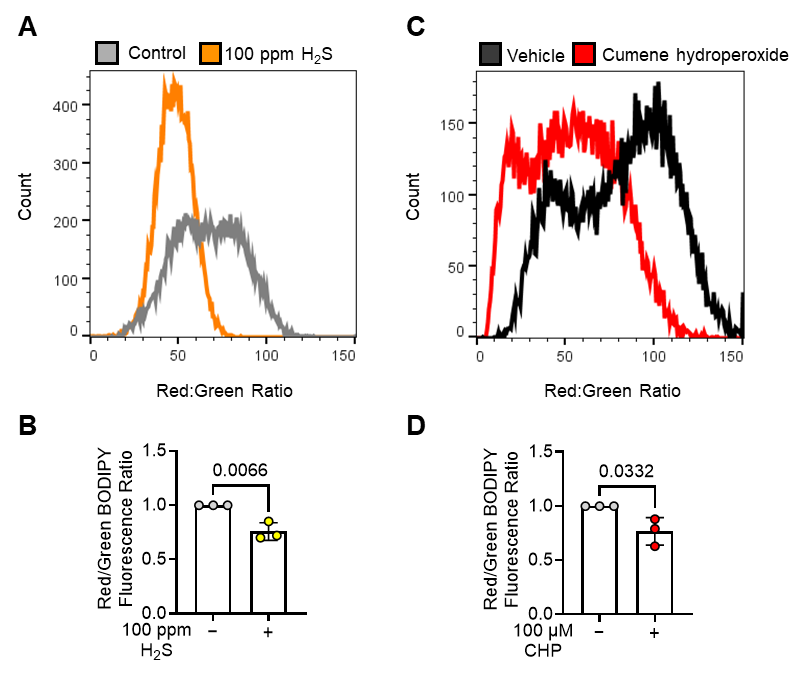


**Supplementary Figure 1: H_2_S increases lipid oxidation**. (A) Ratio of red:green fluorescence, indicating the reduced:oxidized ratio of BODIPY 581/591 C11 lipid peroxidation sensor in HT-29 cells exposed to 100 ppm H_2_S for 24 h. (B) Quantitation of the red/green BODIPY fluorescence ratio from A. (C) Positive control with the same cells exposed to 100 µM cumene hydroperoxide (CHP) to induce lipid ROS. (D) Quantitation of the red/green BODIPY fluorescence ratio from C. An unpaired t-test was performed for statistical analysis and data represent mean ± SD.

**Supplementary Figure 2: HIF destabilization by H_2_S across cell lines**. (A,B) Representative western blots (A) and quantitation (B) of colon UQCRFS1 from Sqor^fl/fl^ (n=3) and *Villin*^Cre^ Sqor^fl/fl^ mice (n=3). (C) HT-29 cells grown in a 2% O_2_ atmosphere were exposed to 300 µM Na_2_S for the indicated times and HIF-1α was detected in cell lysates by western blot analysis. (D) Quantitation of HIF-1α intensity in (C) normalized to Ponceau staining and expressed relative to the sample intensity at 1 h without H_2_S. (E,F) Western blot analysis (E) and quantitation (F) of HIF-2α levels in HT-29 cells in response to 1 h hypoxia ± 300 μM H_2_S normalized to β-actin used as a loading control. (G-I) H_2_S dose dependence of HIF-1α destabilization in (G) EA.hy296, (H) HCT116, and (I) HEK293 cells grown in 2% O_2_. (J-L) Quantitative analysis of western blots for HIF-1α in G-I, respectively normalized to Ponceau staining and presented relative to untreated controls (n=4). Data represent mean ± SD. Statistical analysis was performed with an unpaired t-test (B,F), one-way ANOVA followed by Šídák post-hoc test (J-L), or two-way ANOVA followed by Šídák post-hoc test (D).

**Supplementary Figure 3. H_2_S-induced increase in O_2_ monitored by dUnaG**. (A) Effect of H_2_S (10, 25 and 100 ppm) on dUnaG expression in HT-29 cells grown in 2% O_2_. The images are representative of 3 independent experiments. (B,C) Effect of H_2_S (10, 25 and 100 ppm) on UnaG (B) and dUnaG (C) expression in HEK293 cells grown in 2% O_2_. The images are representative of 3 independent experiments. (D-F) Quantitation of fluorescence intensity in A-C, respectively, normalized relative to the sample lacking H_2_S in each panel. (G,H) Bolus treatment of HEK293 cells expressing UnaG grown in 2% O_2_ with 1 mM H_2_S did not affect fluorescence. Scale bar is 200 μm in all images. Data represent mean ± SD. Statistical analysis was performed with one-way ANOVA followed by Šídák post-hoc test (D-F), or an unpaired t-test (H).

**Supplementary Figure 4: O_2_ calibration curves in HT-29 and HEK cells**. (A-C) Expression of dUnaG in HT-29 (A) or HEK293 (C) cells or UnaG in HEK293 cells (B) grown in 2%-21% O_2_ (without H_2_S). (D-F) Dependence of dUnaG (D, F) and UnaG (E) fluorescence on O_2_ concentrations (normalized to 2% O_2_ =1) and fit to an exponential decay curve (n=3). The green dots correspond to fluorescence observed at the indicated concentration of H_2_S (data shown in D-F are from A-C, respectively). Scale bar is 200 μm in all images. Data represent mean ± SD.

**
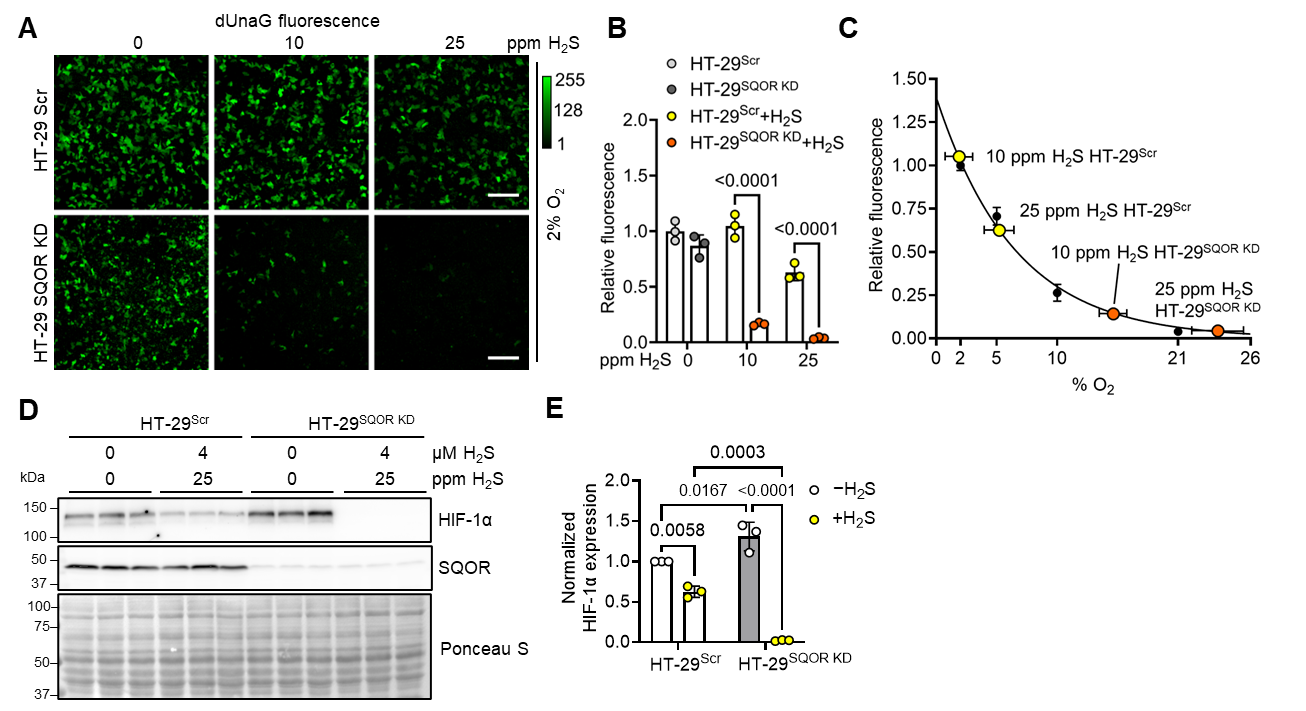
**

**Supplementary Figure 5: SQOR deficiency sensitizes cells to HIF destabilization by H_2_S**. (A) Effect of H_2_S (10 and 25 ppm, 24 h) on dUnaG expression in HT-29^Scr^ and HT-29^SQOR KD^ cells grown in 2% O_2_. The images are representative of 3 independent experiments. Scale bar is 200 μm. (B) Quantitation of F normalized to HT-29^Scr^ without H_2_S. (C) UnaG fluorescence data in B was plotted on the standard curve for dUnaG fluorescence of O_2_ concentration. (D) HIF-1α and SQOR expression in HT-29^Scr^ and HT-29^SQOR KD^ cells ± 25 ppm H_2_S exposure for 24 h. (E) Quantitation of HIF-1α levels in D normalized to Ponceau S staining and shown relative to HT-29^Scr^ levels. Data represent mean ± SD. Statistical analysis was performed with a two-way ANOVA followed by Šídák post-hoc test (B), or Tukey post-hoc test (E).

**Supplementary Figure 6: HIF destabilization by H_2_S is PHD-dependent**. (A) The PHD inhibitor FG4592 was added to HT-29 cells at the indicated concentrations, and HIF-1α was detected by western blotting. (B) HT-29 cells cultured in 21% O_2_ were treated with 300 µM Na_2_S and 30 µM FG4592, and HIF-1α expression was detected by western blotting. (C) Quantitation of HIF-1α levels in B normalized to Ponceau S staining (n=3). (D) HT-29 cells were pre-treated with the proteasome inhibitor bortezomib (1 µM) for 6 h and then cultured in a 2% O_2_ incubator ± 300 µM Na_2_S for 1 h prior to HIF-1α detection. Accumulation of high molecular weight (HMW) bands assigned to ubiquitinated HIF was observed in the bortezomib-treated samples. (E,F) Quantitation of HIF-1α levels in D normalized to Ponceau S staining (E) or total HIF-1α levels (F) (n=4). Data represent mean ± SD. Statistical analysis was performed with unpaired t-tests.

**Supplementary Figure 7: H_2_S does not affect HIF-1α synthesis.** (A) Scheme showing that decreased translation could be an additional target for H_2_S-mediated HIF-1α destabilization. (B) Puromycin (1 μg/mL, 1 h) led to the accumulation of nascent puromycin-containing peptides, which were not seen in untreated HT-29 cells grown at 21% O_2_. The intensity and banding pattern of the nascent puromycin peptides was unaffected by 300 μM H_2_S. A splice line delineating two separate blots is denoted in red. (C) HT-29 cells were grown in 21% O_2_ ± 100 ppm H_2_S for 23 h, then incubated for 1 h under the same conditions with 1 μg/mL puromycin. Puromycin-labeled nascent peptides were detected by western blotting. (D) Quantitation of HIF-1α levels in B normalized to Ponceau S staining (n=4). (E,F) Western blot analysis of eIF2α phosphorylation in HT-29 cells grown in the presence of 100 ppm H_2_S and 21% O_2_ after (E) 1 h and (F) 24 h. (G) Phosphorylation of eIF2α in HT-29 cells grown in 21% O_2_ and exposed to 300 μM H_2_S for varying times. (H) Quantitation of phospho-eIF2α relative to total eIF2α which was normalized to Ponceau S staining (n=3). Data represent mean ± SD.
